# Supplementary material for: Cyclodextrin Metal-Organic Framework as a Broad-Spectrum Potential Delivery Vehicle for the Gasotransmitters
Source: Molecules. 2023 Jan 14;28(2):852. doi: 10.3390/molecules28020852 (PMC9866194; doi:10.3390/molecules28020852)
Supplement: Supplementary file 1 [file molecules-28-00852-s001.zip › molecules-2114758-supplementary.pdf]

Supplementary Materials

Cyclodextrin Metal-Organic Framework as a Broad-Spectrum Potential Delivery Vehicle for the Gasotransmitters

Li-Guo Liao [1,2,†], Duo Ke [1,2,†], Guo-Chen Li [3], Sheng Zhang [3,*] and Bang-Jing Li [1,2,*]

[1] Key Laboratory of Mountain Ecological Restoration and Bioresource Utilization, Chengdu Institute of Biology, Chinese Academy of Sciences, Chengdu 610041, China
[2] University of Chinese Academy of Sciences, Beijing 100049, China
[3] State Key Laboratory of Polymer Materials Engineering, Polymer Research Institute of Sichuan University, Chengdu 610065, China
* Correspondence: zslbj@163.com (S.Z.); libj@cib.ac.cn (B.-J.L.)
† These authors contributed equally to this work.

1. Experimental Section

1.1. Synthesis of UiO-66 and UiO-66-(OH) [1]

$ZrCl_4$ (125 mg, 0.54 mmol), 5 mL dimethyl formamide (DMF) and 1 mL HCl were loaded and sonicated for 10 min until all solids fully dissolved. Then, terephthalic acid (135 mg, 0.75 mmol) and 10 mL DMF were added to the solution and sonicated for more 20 min. The obtained mixture was placed in an oven and heated at 80 °C for 12 h. After cooling down to room temperature, the resulting solid was filtered, repeatedly washed several times with DMF and then with ethanol. Finally, it was dried at 120 °C under vacuum to obtain the MOF. The synthesis procedure of UiO-66-$(OH)_2$ was consistent that for UiO-66, except that terephthalic acid was replaced by 2-hydroxyterephthalic acid.

1.2. Cytotoxicty assays

Mouse fibroblast cells (ATCC CCL1, NCTC Clone 929, Clone of Strain L) were proliferated at 37 ℃, 5% CO_2 in petri dishes containing MEM (HyClone™) supplemented with 10% fetal bovine serum and antibiotics (100U mL^{-1} penicillin, 100μg mL^{-1} streptomycin). After grew nearly confluent, the cells were trypsinized, collected and adjusted to 1×10^5 cells mL^{-1} for experiment.

Mouse fibroblast cells were seeded in 96-well plate with 1×10^4 cells $well^{-1}$, incubated at 37 ℃, 5% CO_2 for 24 hours. Then the supernatant was discarded, and 100μL of the samples (containing 10% FBS) with different dosage was added. MEM (containing 10% FBS) were used as blanks control, the extract liquid of polystyrene as negative control and 10% DMSO as positive control. After cultured at 37 ℃, 5% CO_2 for 24 hours, the supernatant was discarded, and 50 μL MTT solution (1mg mL^{-1}) was added to each well, the plates were incubated for 2 hours and the absorbency was measured at 570nm by a microplate reader. The cell viability was calculated by comparing with the following equation:

$$Cell\ viability = OD_{sample} / OD_{control}$$

Where OD_{sample} is the absorbancy of the cells incubated with samples and where $OD_{control}$ is the absorbancy of the cells incubated without the sample.

1.3. Calculation of Surface Coverage of -OH Groups

The concentration of surface–OH groups in γ-CD-MOF has been calculated.

[K (OH) $(C_{48}H_{80}O_{40})_{0.5}$] Mt = 704.67; BET = 778 $m^2\ g^{-1}$ = 778 x 10^{18} (100 $Å^2\ g^{-1}$)

1/704.67 ×6.02 × 10^{23} / (778× 10^{18}) = 1.098 OH/100 $Å^2$

Table S1. The C/O atomic ratios on γ-CD-MOF surface.

Sample	C (Atomic, %)	O (Atomic, %)	C/O
γ-CD-MOF	59.64	40.09	1.49
γ-CD-MOF-L31	64.61	34.30	1.88
γ-CD-MOF-L63	65.52	33.74	1.94

Table S2. Comparison of SO_2 load and release rate from different pluronics modified γ-CD-MOF.

materials	$M_{w\ (Pluronics)}$	Loading capacity (μmol mg^{-1})
γ-CD-MOF-L31	1100	0.56
γ-CD-MOF-L63	2650	0.51

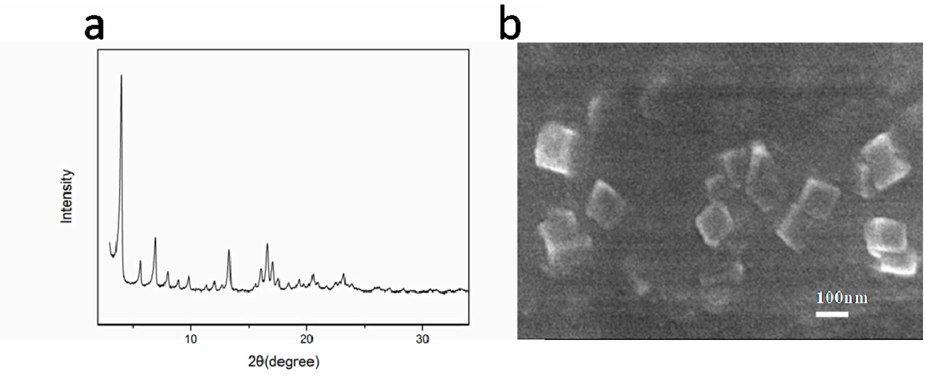

Figure S1. (a) PXRD crystallinity patterns for γ-CD-MOF;

(b) SEM image of γ-CD-MOF.

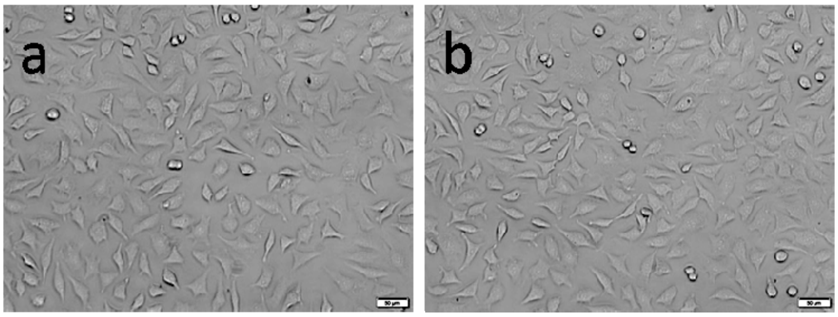

Figure S2. Microscope images of (a) cell culture; (b) cell culture after 24h in contact with γ-CD-MOF (1.2mg ml^{-1}).

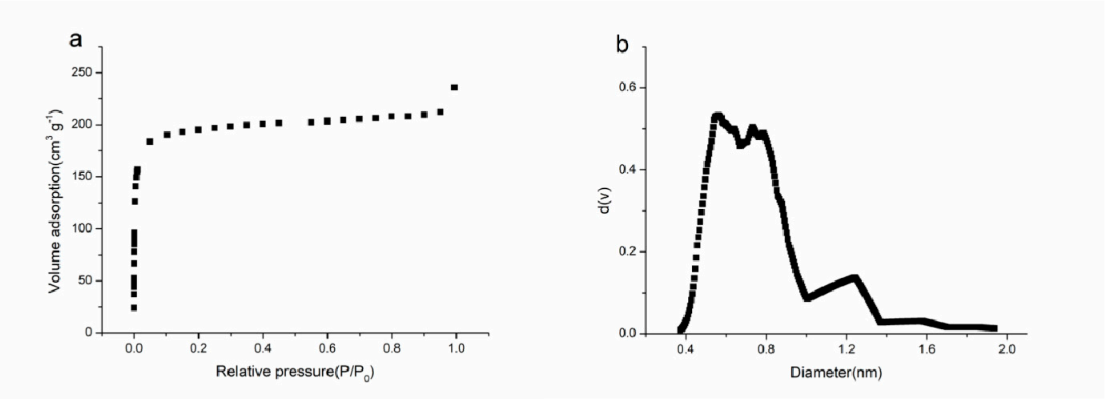

Figure S3. (a) N_2 sorption isotherms at 77K of γ-CD-MOF; (b) Pore size distributions of γ-CD-MOF analyzed with Horváth-Kawazoe (HK) method from N_2 adsorption isotherms at 77K.

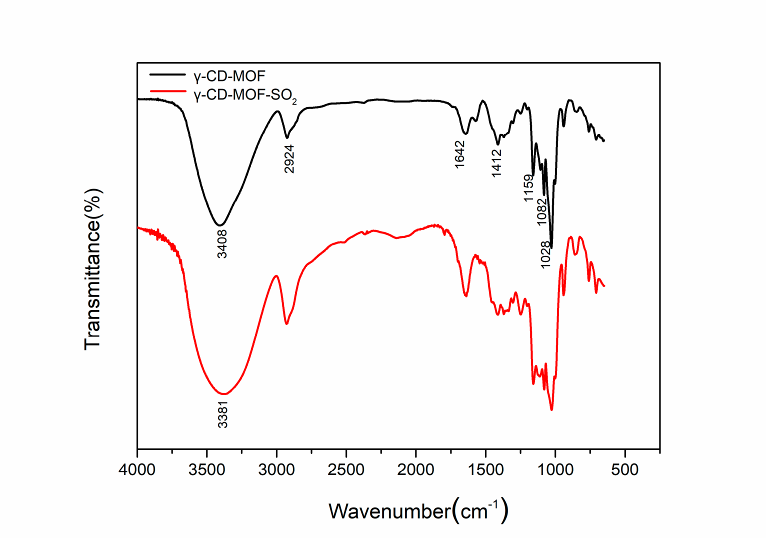

Figure S4. The IR spectrum of γ-CD-MOF and γ-CD-MOF-SO_2.

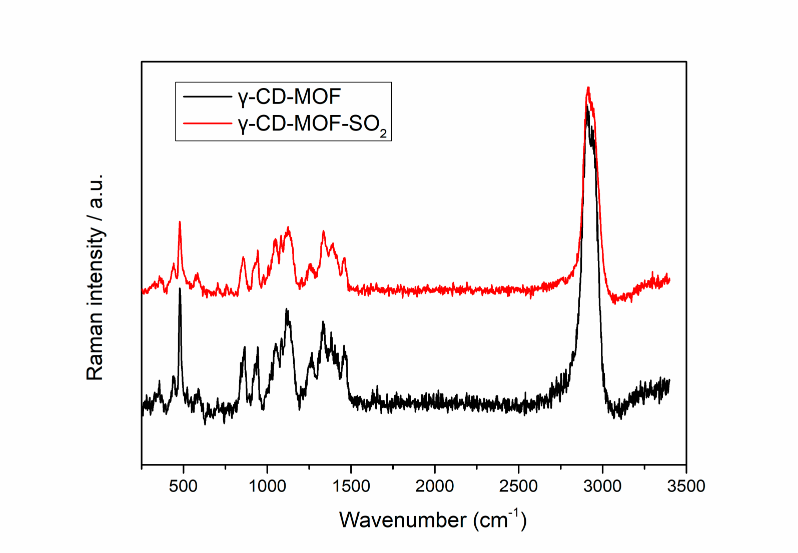

Figure S5. The Raman spectrum of γ-CD-MOF and γ-CD-MOF-SO_2.

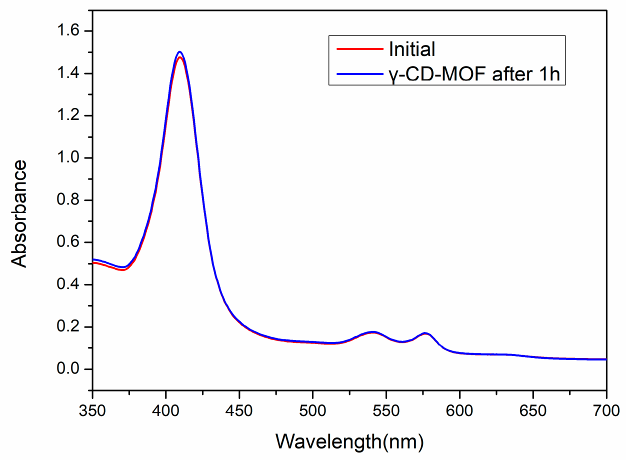

Figure S6. The UV/Vis spectrum of oxyhemoglobin solution upon introduction of γ-CD-MOF (not loaded with NO).

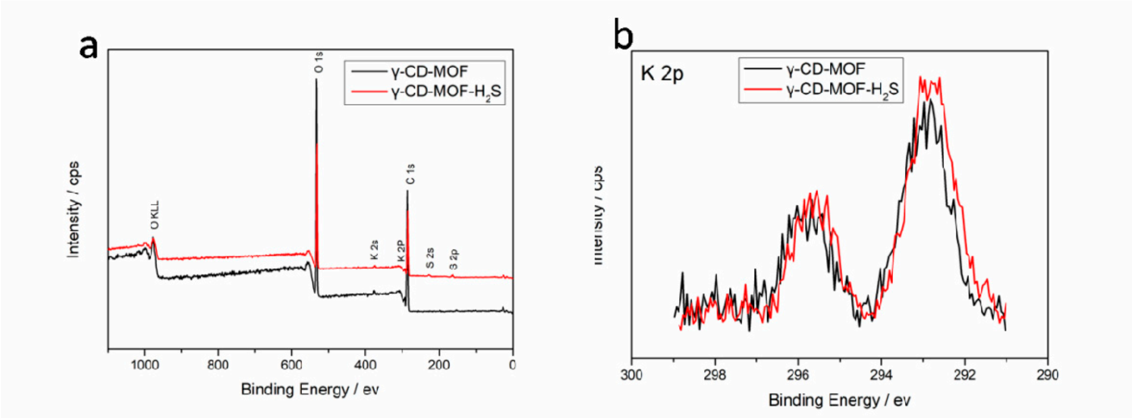

Figure S7. (a) XPS survey spectra of γ-CD-MOF and γ-CD-MOF loaded with H_2S; (b) XPS spectra of K 2p spectra for γ-CD-MOF and γ-CD-MOF-H_2S.

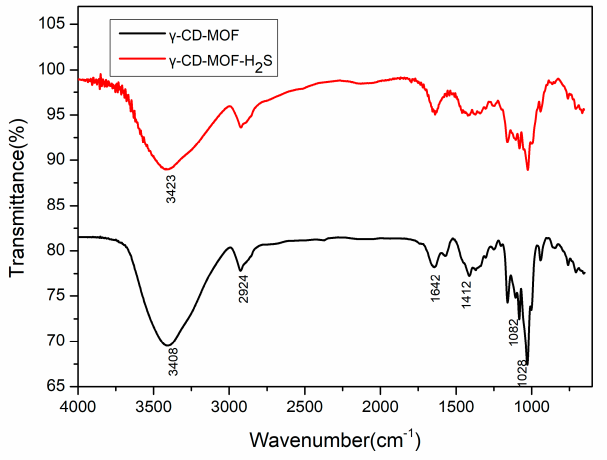

Figure S8. The IR spectrum of γ-CD-MOF and γ-CD-MOF-H_2S.

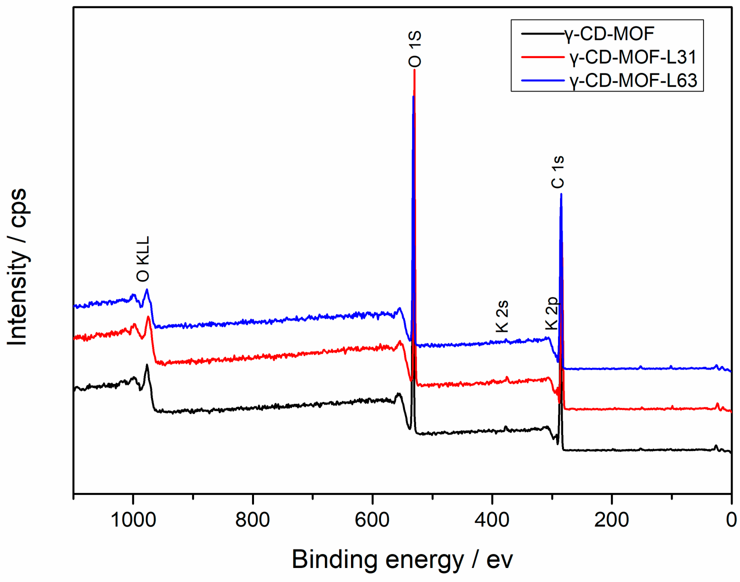

Figure S9. XPS spectra of γ-CD-MOF and different pluronics modified γ-CD-MOF.

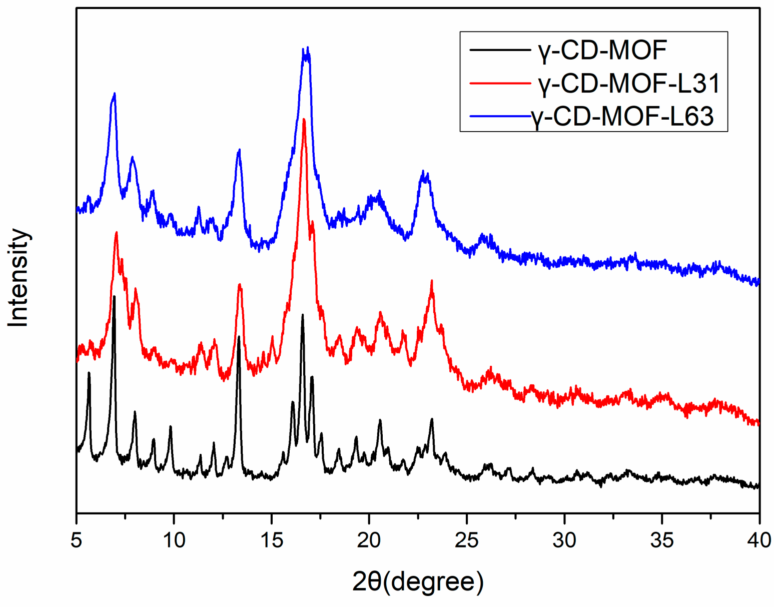

Figure S10. PXRD crystallinity patterns of γ-CD-MOF and different Pluronics modified γ-CD-MOF.

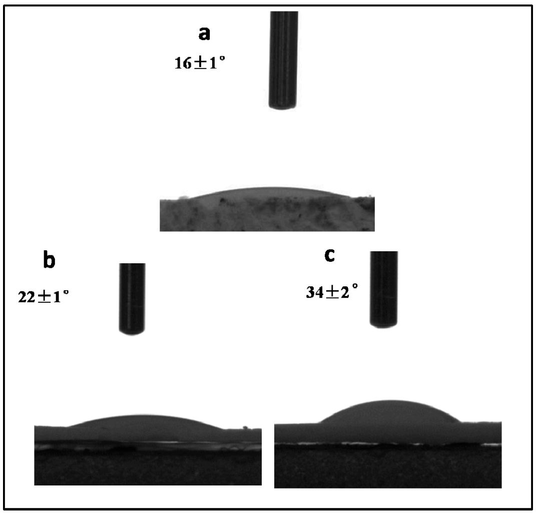

Figure S11. The water contact angel of (a) γ-CD-MOF; (b) γ-CD-MOF-L31; (c) γ-CD-MOF-L63.

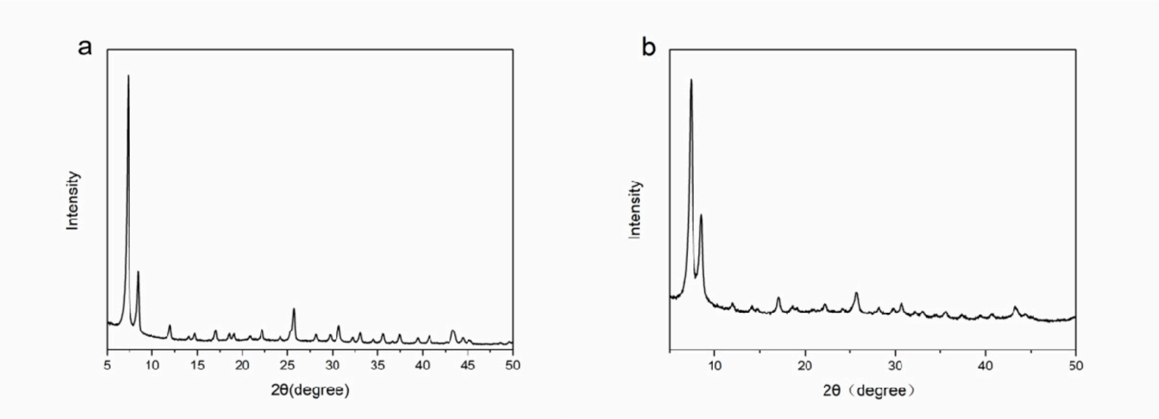

Figure S12. PXRD crystallinity patterns (a) UiO-66; (b) UiO-66-$(OH)_2$.

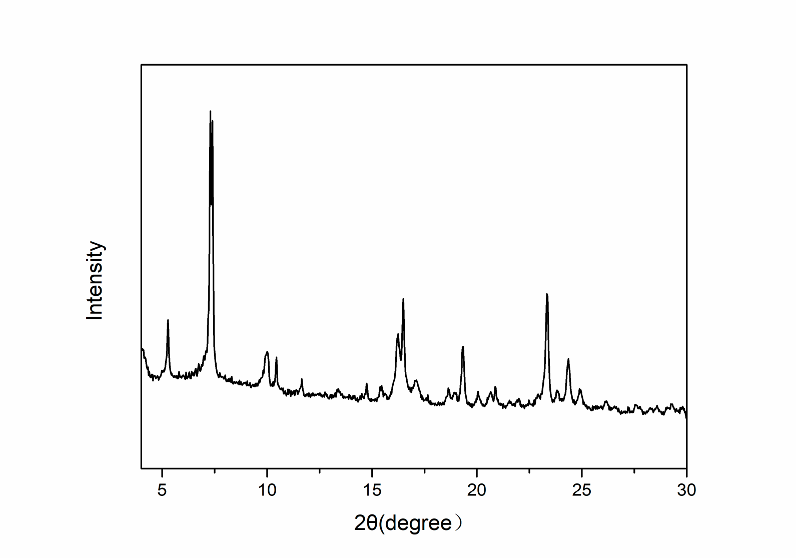

Figure S13. PXRD crystallinity patterns of γ-CD-MOF-Cs.

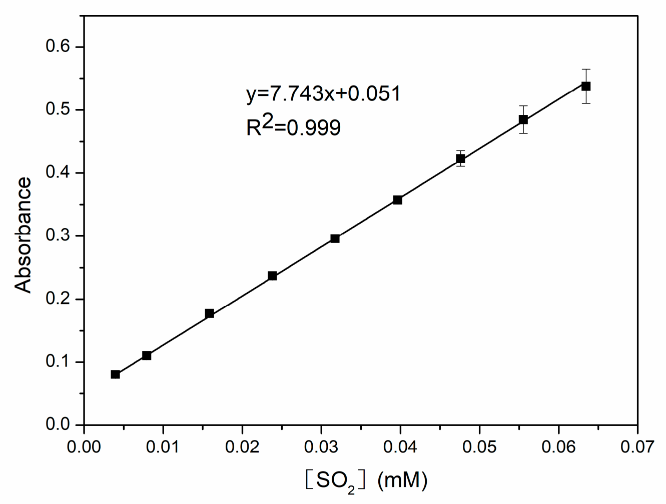

Figure S14. Calibration curve for SO_2 in phosphate buffer containing 1.0 mM EDTA (PH=7.4).

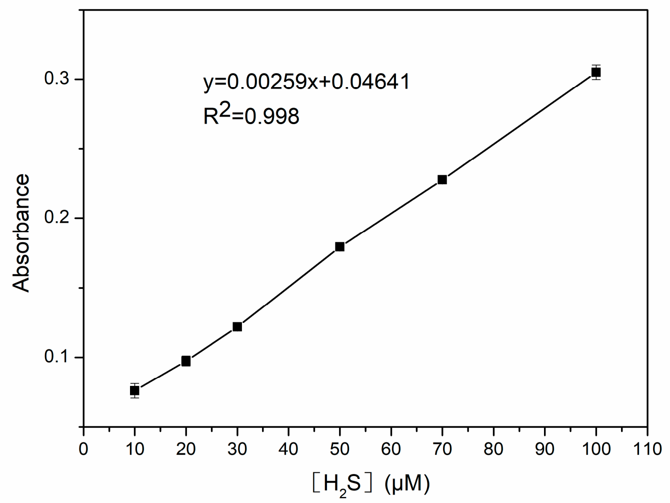

Figure S15. Calibration curve for H_2S in phosphate buffer (PH=7.4).

References

[1] Michael, J. K.; Zachary, J. B.; Yamil, J. C.; Paul, W. S.; Karl, A. S.; Randall, Q. S.; Joseph, T. H.; Omar, K. F. A facile synthesis of UiO-66, UiO-67 and their derivatives. *Chem. Commun.* **2013**, *49*, 9449-9451.
